# Oxidative stress in refractory and recurrent vitiligo: from immune amplification mechanisms to potential stratified antioxidant combination therapy

**DOI:** 10.3389/fimmu.2026.1978208

**Published:** 2026-09-25

**Authors:** Hao Xu, Qing Yang, Rong Jin, Wenting Hu, Wen Xu, Aie Xu

**Affiliations:** 1 Department of Dermatology, Hangzhou Third People’s Hospital, Hangzhou, China; 2 School of Pharmacy, Shanghai Jiao Tong University, Shanghai, China; 3 Yunnan Characteristic Plant Extraction Laboratory Co., Ltd., Yunnan, Kunming, China

**Keywords:** antioxidant therapy, combination therapy, oxidative stress, recurrent vitiligo, refractory vitiligo

## Abstract

Vitiligo is an acquired depigmenting disorder characterized by destruction of epidermal melanocytes and skin depigmentation. Although treatments have developed substantially, there is still a considerable proportion of vitiligo patients fail to achieve satisfactory repigmentation or experience disease recurrence after treatment withdrawal. Vitiligo treatment failure involves heterogeneous clinical states, including persistent disease activity, treatment resistance, site-related limitations, and disease recurrence. Importantly, refractory and recurrent vitiligo are heterogeneous and partially overlapping clinical issues rather than a single biological damage requiring a uniform management strategy. Oxidative stress serves as a critical factor in the pathogenesis of vitiligo, linking melanocyte injury, release of danger-associated molecular patterns (DAMPs), innate immune activation, and IFN-γ driven adaptive immune amplification, associated with active and refractory disease. Interleukin (IL)-15-dependent tissue-resident memory T (TRM) cells provide a mechanism for local immune reactivation and recurrence. However, the role of oxidative stress in treatment resistance and recurrence remains incompletely defined. Importantly, available studies of antioxidant therapy are heterogeneous, with no evidence demonstrates that antioxidant therapy eliminates TRM cells or prevents recurrence. In this review, we propose a hypothesis-generating clinical framework that distinguishes refractory and recurrent vitiligo based on disease activity, IFN-γ signaling, anatomical sites, phototherapy responses and recurrence risk. Currently, antioxidant therapy should be considered as an investigational adjunctive therapy. The review investigates the relationship between oxidative stress and antioxidant therapy in refractory and recurrent vitiligo, and evaluates the role of antioxidant therapy in the management of vitiligo. In the future, it will be essential to conduct prospective biomarker based stratified clinical trials to define the targeted vitiligo patients for antioxidant combination therapies, as well as to continuously develop novel antioxidant-based anti-inflammatory therapeutic approaches for refractory and recurrent vitiligo.

## 1 Introduction

Vitiligo is an acquired autoimmune pigmentary disorder characterized by loss of epidermal melanocytes and skin depigmentation, affecting approximately 0.36% of the population worldwide (1–3). Vitiligo causes a significant disease burden, with patients experiencing more immune-related comorbidities, psychological effects, and impaired quality of life (1, 3, 4). A central effector pathway in vitiligo is the induction of chemokines by IFN-γ signaling, which recruits cytotoxic T cells to attack melanocytes (5, 6). However, the cause of vitiligo is complex, resulting from the combined effects of genetic susceptibility, oxidative stress, innate and adaptive immune activation, and dysregulation of the immune microenvironment (1).

Many studies have demonstrated that exogenous triggers such as ultraviolet radiation (UVA and UVB), and chemical exposure (like phenols and quinols) directly induce melanocyte damage and reactive oxygen species (ROS) generation, preceding immune-mediated melanocyte destruction (1, 7–11). Recent studies suggest that the stress response and dysfunction of melanocytes typically precede significant immune-mediated destruction, thereby converting stressed melanocytes into immunogenic targets (1, 7, 8, 12). Under oxidative stress conditions, melanocytes can be activated through endoplasmic reticulum (ER) stress, unfolded protein response (UPR) pathways, subsequently releasing cytokines, chemokines, senescence-associated secretory phenotype (SASP), and melanocyte-derived autoantigens, inducing the maturation of dendritic cells (DCs) and the recruitment and activation of CD8^+^ T cells (1, 7, 8, 12–19). Therefore, the progression of vitiligo involves not only the destruction of melanocytes, but also the activation of innate and adaptive immunity by melanocytes under oxidative stress, transforming localized redox imbalance into a sustained immune response that drives vitiligo progression. Based on the important role of oxidative stress and potential value and efficacy of antioxidant therapy in vitiligo, antioxidant therapy may be considered for combination with other therapeutic strategies to treat vitiligo (10, 20).

Nowadays, management goals for vitiligo have shifted from the traditional single-treatment approach focused on disease control and appearance improvement to a modern management paradigm that emphasizes early identification of active disease, long-term disease management, individual comprehensive therapy, and recurrence reduction (20–23). Topical corticosteroids, calcineurin inhibitors, Janus kinase (JAK) inhibitors, narrowband ultraviolet B (NB-UVB) phototherapy, 308-nm excimer laser, and surgical melanocytes or melanocyte stem cells transplantation each have distinct therapeutic effects (20–23). However, therapeutic efficacy varies significantly depending on anatomical site, disease duration, disease activity, and follicular melanocyte reserve (24). Lesions on the face and neck typically respond well, whereas those on the acral and joint sites, bony prominences, lip, and white hair show poorer responses (25–28). Of concern is that the recurrence rate of vitiligo lesions reaches approximately 44% during the first year after discontinuation of maintenance therapy (1, 21, 29). Therefore, achieving durable repigmentation remains challenging, particularly for patients with persistent disease activity or recurrent relapses after treatment.

Nevertheless, the concept of treatment failure in vitiligo involves heterogeneous clinical problems that should be mechanistically distinguished to improve repigmentation in refractory lesions and prevent recurrence after treatment. Refractory vitiligo generally refers to lesions that fail to achieve satisfactory repigmentation and remain resistant to adequate treatment (20, 24, 30). In contrast, recurrent vitiligo occurs after successful repigmentation and is associated with the persistence of autoimmune memory, particularly with skin resident memory T (TRM) cells (29, 31–33). These problems have two partially distinct but overlapping biological processes. Refractory vitiligo is more closely associated with anatomical site, sustained IFN-γ and chemokine-driven inflammation, insufficient melanocyte reserves in hair follicles, or treatment resistance (24, 26, 27). Recurrent vitiligo is increasingly related to interleukin (IL)-15-CD122-TRM axis that persists within clinically repigmented skin (31, 34, 35). Refractory and recurrent vitiligo should be distinguished in terms of treatment strategies, with a focus on exploring the potential role of antioxidant therapy in improving treatment response and supporting long-term disease management.

Accordingly, this review does not simply summarize antioxidant therapy in vitiligo. Instead, we discuss how oxidative stress interacts with immune pathways throughout the vitiligo progression and evaluate whether antioxidant therapy may serve as the complementary treatment to improve therapeutic outcomes, particularly in the management of refractory and recurrent vitiligo.

## 2 Redox homeostasis and pathological oxidative stress in vitiligo

Reactive oxygen species (ROS) are physiological signaling molecules as well as potential mediators of cellular injury (36, 37). Melanogenesis is intrinsically associated with ROS generation, making melanocytes susceptible to oxidative stress (36, 38). Under physiological conditions, small amounts of ROS are converted into non-toxic substances by the antioxidant system (7). At low levels, ROS play an important role in melanogenesis, melanocyte signaling, and UV-induced biological responses (39–41). Experimental results indicate that low-dose ROS play a major role in promoting melanin synthesis and melanosome transfer (39). Therefore, the presence of ROS should not be regarded as pathological. However, oxidative stress arises when ROS generation exceeds the capacity of endogenous antioxidant and redox systems. Evidence from patients with vitiligo supports the presence of dysregulated redox homeostasis. In the skin of patients with vitiligo, elevated levels of ROS have been consistently observed, affecting melanocytes (1). Increased ROS production and impaired antioxidant defense shift this balance towards persistent oxidative stress in vitiligo progression (1, 7, 10). A previous clinical study found that serum levels of catalase (CAT), superoxide dismutase (SOD), and total antioxidant capacity (TAC) were significantly reduced, while lipid oxidation product malondialdehyde (MDA) and 8-hydroxy-2-deoxyguanosine (8-OHdG) were markedly elevated in patients with non-segmental vitiligo (NSV), indicating diminished antioxidant defense capacity accompanied by significant lipid and DNA oxidative damage. Among these markers, TAC, MDA, and 8-OHdG were correlated with disease activity and severity, with 8-OHdG potentially serving as a valuable serum biomarker for assessment (42). However, these findings should not now be interpreted as defining a universal biochemical phenotype or a clinically validated threshold of oxidative stress in vitiligo. These biomarkers may provide a basis for future patient stratification, although they require prospective validation.

When ROS production becomes excessive or antioxidant responses are insufficient, it leads to impairing melanocyte survival and function (10). Excessive ROS result in melanocyte damage and dysfunction through multiple mechanisms, including endoplasmic reticulum (ER) stress, mitochondrial dysfunction, adhesion defects, and various immune and inflammatory pathways related to vitiligo progression (7). Oxidative stress dynamically interacts with intrinsic defects of melanocytes, innate immune activation, and adaptive immune responses throughout vitiligo progression (1, 7, 8, 10). Cellular stress transforms melanocytes from attacked targets into active roles in immune activation (1, 7, 13). Oxidative stress in melanocytes of vitiligo promotes the release of damage-associated molecular patterns (DAMPs), including mitochondrial DNA (mtDNA), high mobility group box-1 protein (HMGB1), and inducible heat shock protein 70 (HSP70i), which activate dendritic cells (DCs) to secrete chemokines and directly induce apoptosis of melanocytes (43, 44). Besides, stressed cells develop a senescence-associated secretory phenotype (SASP) consisting of growth factors, chemokines, and cytokines which recruits immune cells to attack melanocytes (7, 10, 44). More importantly, cytotoxic T lymphocytes (CTLs) secrete IFN-γ to attack melanocytes, which further stimulates melanocytes to release HSP70i, thereby forming a positive feedback loop of HSP70i-Langerhans cells (LCs)-CTLs-IFN-γ-HSP70i that continuously amplifies the local immune response and sustains melanocyte destruction in vitiligo (8, 16, 17). Thus, oxidative stress and immune activation are better viewed as interconnected processes rather than strictly sequential events.

Endogenous antioxidant pathways provide an important defense against excessive oxidative stress. Under physiological stress conditions, accumulated ROS activate nuclear factor erythroid 2-related factor 2 (Nrf2) and this antioxidant response pathway helps prevent oxidative stress and cellular damage (45). In vitiligo melanocytes this pathway and its downstream responses are insufficient, so oxidative stress causes progressively greater cellular damage (46, 47). The redox balance and susceptibility to vitiligo patients are highly correlated with Nrf2, and the Nrf2 pathway can modulate the dynamic balance of ROS and suppress the progression of inflammation by regulating the expression of anti-inflammatory genes (48–51). Therefore, in addition to eliminating excessive ROS, it is also essential to activate endogenous antioxidant capacity and suppress downstream immune and inflammatory responses. From the therapeutic perspective, the objective of antioxidant treatment is to reduce excessive oxidative burden and restore redox homeostasis. At present, no validated quantitative ROS threshold defines an optimal therapeutic target in vitiligo, and current evidence does not support antioxidant therapy as an independent treatment. The antioxidant therapy should therefore be considered investigational adjuncts to established therapies.

Collectively, oxidative stress represents an important component of vitiligo pathogenesis by impairing melanocyte homeostasis, promoting stress-associated immunogenic signals, and interacting with innate and adaptive immune responses. However, oxidative stress is not a single cause of vitiligo or an isolated event preceding immune activation, and redox dysregulation and immune inflammation may coexist during disease progression. Therefore, antioxidant therapy requires combination with immune-targeted therapies (**Figure 1**).

**Figure 1:**
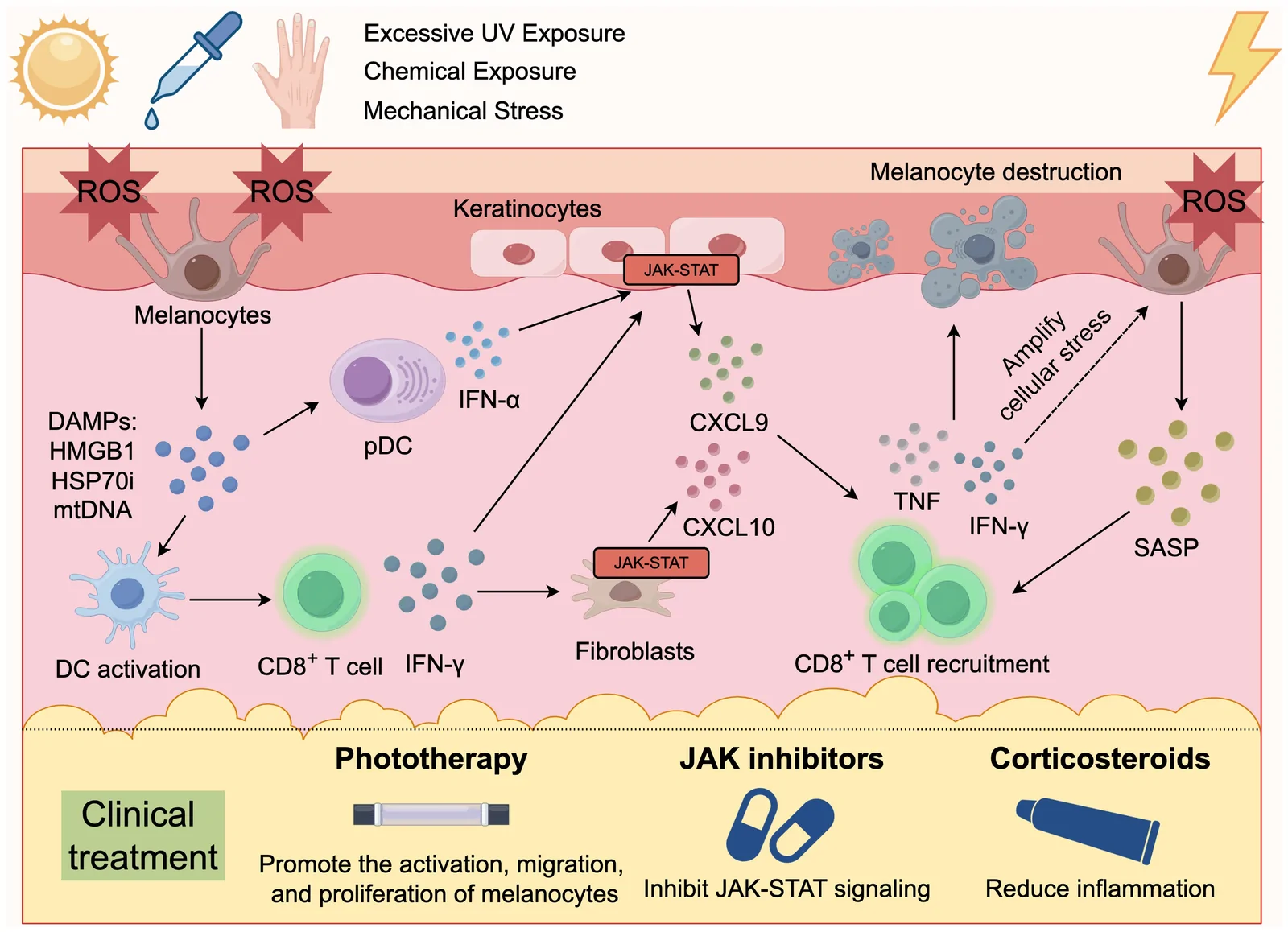
Oxidative stress in vitiligo development. Environmental stress including UV and chemical exposure, mechanical stress induces melanocyte damage and ROS generation. Oxidative stress in melanocytes of vitiligo promotes the release of DAMPs, inducing pDCs to produce IFN-α and DCs to activate autoreactive CD8^+^ T cells which subsequently produce IFN-γ. IFN-α/γ-responsive keratinocytes and IFN-γ-responsive fibroblasts produce CXCL9 and CXCL10 through JAK-STAT signaling to recruit CD8^+^ T cells that produce IFN-γ and TNF leading to melanocyte destruction or amplifying cellular stress. Besides, stressed cells develop SASP which recruits T cells to attack melanocytes. Phototherapy promotes the activation, migration, and proliferation of melanocytes, JAK inhibitors interrupt JAK-STAT signaling, and corticosteroids suppress inflammation. UV, ultraviolet radiation; ROS, reactive oxygen species; DAMPs, damage-associated molecular patterns; pDCs, plasmacytoid dendritic cells; IFN, interferon; DCs, dendritic cells; CXCL, C-X-C motif ligand; JAK, Janus kinase; STAT, signal transducer and activator of transcription; TNF, tumor necrosis factor; SASP, senescence-associated secretory phenotype. Created by FigDraw.

## 3 From oxidative stress to immune persistence in vitiligo: two distinct but overlapping circuits

Two overlapping immunological pathways respectively determine whether the disease remains active and refractory, as well as whether it recurs subsequently. Persistent IFN-γ signaling amplifies inflammation and maintains an active state, which is a major contributor to disease refractoriness (52). Meanwhile, the long-term presence of TRM cells in the skin sustains local immune memory, leading to vitiligo recurrence (24, 34). These two circuits represent the underlying mechanisms of refractory vitiligo and recurrent vitiligo respectively, and also guide distinct therapeutic strategies for each condition.

The IFN-γ-C-X-C motif chemokine ligands (CXCL) 9/10-C-X-C motif chemokine receptor (CXCR) 3 axis drives active, chemokine-amplified vitiligo (53). Oxidative stress-induced melanocytes release DAMPs, inducing dendritic cells to activate autoreactive melanocyte-specific CD8^+^ T cells, which subsequently produce IFN-γ (1, 7). Through activation of the Janus kinase (JAK)-signal transducer and activator of transcription (STAT) signaling pathway in keratinocytes and fibroblasts, IFN-γ induces robust expression of CXCL9 and CXCL10 (6, 52, 54). These chemokines recruit CXCR3 positive cytotoxic CD8^+^ T cells into lesional skin, establishing a positive amplifying loop that promotes melanocyte destruction and sustains vitiligo activity (53, 55). The deeper understanding of the central role of the IFN-γ signaling pathway in vitiligo has facilitated advancements in vitiligo treatment and the development of targeted therapies. The mechanism of JAK inhibitors (JAKi) focuses on inhibiting the IFN-γ-mediated JAK-STAT signaling pathway, reducing the production of downstream chemokines (53). The topical JAK inhibitor ruxolitinib cream 1.5% is now the first FDA and EMA approved treatment for vitiligo (non-segmental), and clinical trials have demonstrated that the score of the vitiligo area assessment index shows significant and durable improvement after treatment (56, 57). Beyond topical ruxolitinib, accumulating evidence suggests that JAKi represent a broader therapeutic strategy in vitiligo rather than a drug-specific effect. Tofacitinib in combination with NB-UVB phototherapy has shown potential efficacy in refractory vitiligo treatment, although the available evidence is limited (58). Oral ritlecitinib was effective for patients with active non-segmental vitiligo in a randomized phase 2b clinical trial (59). Besides, upadacitinib and Janus kinase 1 inhibitor povorcitinib demonstrated substantial total body and facial repigmentation in adult patients with extensive non-segmental vitiligo (60, 61). These findings collectively indicate the therapeutic relevance of JAK-mediated signaling in vitiligo, although most systemic JAKi remain investigational and require further assessment of long-term efficacy and safety. Importantly, oxidative stress and IFN-γ-JAK-STAT-mediated immune activation should not be regarded as strictly sequential or discrete events. By the time depigmented lesions are clinically present, oxidative stress and immune amplification may occur concurrently. Antioxidants cannot therefore be assumed to prevent pathway initiation, but can be an adjunct to therapies that suppress the established immune circuit and promote repigmentation. However, the clinical efficacy, optimal timing, and potential synergy of this combination have not yet been established.

TRM cells are formed in the lesions of vitiligo patients, contributing to disease recurrence (31, 33, 62). In the murine vitiligo model, JAKi can treat vitiligo but do not eliminate TRM cells in the skin (63). Vitiligo-associated CD8^+^ TRM cells are present in both stable and active vitiligo lesions as well as the surrounding skin, and are commonly characterized by expression of CD69 and CD103, with many cells also expressing CXCR3 and the interleukin (IL)-15 receptor β-chain (CD122), and CD49a identifies a more cytotoxic TRM subset (31, 33, 64). Upon stimulation, TRM cells rapidly produce high levels of IFN-γ and TNF, reactivating the JAK-STAT signaling pathway to regulate melanocyte adhesion, mitochondrial metabolism, and a pro-inflammatory state at the local lesion site, thereby sustaining the disease progression (1, 31, 33, 64). IL-15 exerts its effects through its receptor CD122 and is essential for the development, activation, and function of TRM cells (35). TRM cells formation is impaired in IL-15 deficient mice and IL-15 promotes TRM cells functionality *in vitro* (31). In mice with vitiligo, short-term experimental blockade of the IL-15 signaling pathway inhibits IFN-γ production by TRM cells and induces pigmentation, whereas long-term treatment depletes TRM cells in skin lesions, leading to sustained disease reversal (31). Importantly, a completed double blind, placebo-controlled phase 2a trial evaluating AMG 714 for vitiligo has reported results on the trial registry (NCT04338581, https://www.clinicaltrials.gov/; registered April 8, 2020), and the peer-reviewed publication of the clinical findings remains awaited. Although clinical translation remains uncertain, the IL-15/CD122-TRM axis may become one of the most promising therapeutic targets for recurrence prevention rather than a purely preclinical hypothesis. There is currently a lack of direct evidence demonstrating that oxidative stress is essential for maintaining the long-term therapeutic response in human vitiligo. A recent study indicates that oxidative stress increases IL-15 expression and trans-presentation in keratinocytes, causing activation and expression of cytotoxic proteins of CD8^+^ effector memory T (TEM) cells via JAK-STAT signaling in vitiligo (65). This study establishes a mechanistic link between oxidative stress and IL-15-dependent activation of CD8^+^ TEM cells, but it does not directly demonstrate that oxidative stress activates, maintains, or supports the long-term survival of skin TRM cells. Antioxidant therapy may reduce oxidative stress signals and the trans-presentation of IL-15 in keratinocytes, but there is currently no evidence that they can eliminate TRM cells or prevent vitiligo recurrence independently (**Figure 2**).

**Figure 2:**
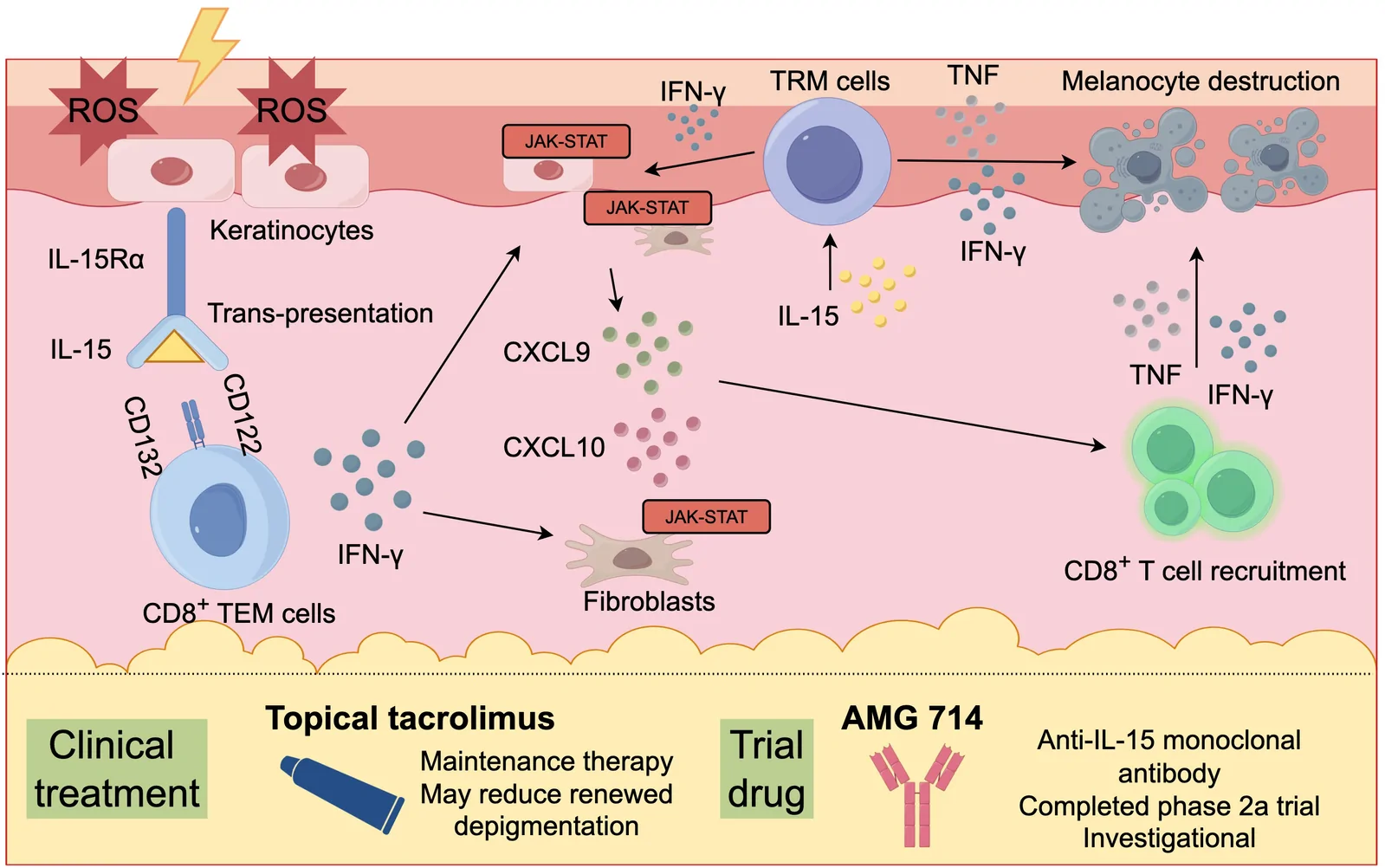
Oxidative stress in recurrent vitiligo. Oxidative stress increases IL-15 expression and trans-presentation in keratinocytes, causing activation of CD8^+^ TEM cells to produce IFN-γ. Upon IL-15 signaling stimulation, TRM cells rapidly produce high levels of IFN-γ and TNF leading to vitiligo recurrence. IFN-γ-responsive keratinocytes and fibroblasts produce CXCL9 and CXCL10 through JAK-STAT signaling to recruit CD8^+^ T cells that produce IFN-γ and TNF leading to melanocyte destruction. Topical tacrolimus may reduce renewed depigmentation, and AMG 714 is an investigational anti-IL-15 monoclonal antibody evaluated in a completed phase 2a trial and the registry results have been posted. IL, interleukin; TEM, effector memory T cells; IFN, interferon; TRM, tissue-resident memory T cells; TNF, tumor necrosis factor; CXCL, C-X-C motif ligand; JAK, Janus kinase; STAT, signal transducer and activator of transcription. Created by FigDraw.

Collectively, the IFN-γ-JAK-STAT-CXCL9/10-CXCR3 signaling pathway and the IL-15/CD122-TRM circuits should be distinguished to guide targeted therapies for refractory and recurrent vitiligo. Combination therapy may hold greater potential: reducing cellular stress through antioxidant therapy, controlling persistent immune amplification with JAKi, and targeting IL-15/CD122 to prevent vitiligo recurrence.

## 4 Antioxidant therapy: clinical positioning in combination therapies

The treatment for vitiligo mainly focuses on suppressing abnormal immune states and promoting the production of melanin. Topical or oral corticosteroids are recommended for vitiligo and suppress immune responses and reduce inflammation to prevent the spread of white patches (20, 66, 67). Topical calcineurin inhibitors suppress abnormal immune responses and maintenance treatment to prevent recurrence (68). Phototherapy is still an important treatment for vitiligo and aims to stimulate the activity of melanocytes and promote pigment regeneration (20, 69). Surgical intervention helps supplement the source of melanocytes or melanocyte stem cells in vitiligo lesions (24, 70). JAKi have emerged as an important immune targeted strategy in vitiligo by suppressing cytokine signaling and reducing the inflammatory environment to create conditions for repigmentation (56, 71). For recurrent vitiligo, in the Topical Ruxolitinib Evaluation in Vitiligo (TRuE-V) long-term extension study, patients who continued JAKi ruxolitinib cream were significantly more likely to maintain F-VASI75 than those undergoing treatment withdrawal, while most patients experiencing recurrence after discontinuation successfully regained pigmentation upon treatment re-initiation (72, 73). An earlier randomized, double-blind, placebo-controlled trial established that intermittent topical tacrolimus reduced the risk of depigmentation recurrence in previously repigmented lesions (32). Maintenance therapy may increasingly shift towards IL-15-dependent survival of TRM cells, one of the potential explanations for recurrence, however, antioxidant therapy is unlikely to influence the survival of TRM cells directly. Current evidence does not support antioxidant therapy as an effective treatment alone for vitiligo. Many studies indicate that combining antioxidant therapy with NB-UVB phototherapy, they can achieve their maximum therapeutic efficacy. Therefore, antioxidant therapy should be regarded as an adjunctive therapy for oxidative stress in vitiligo progression rather than a primary treatment strategy. Clinical data on antioxidant therapy for refractory vitiligo are limited, and there is no consensus on the therapeutic effect. Future research should prioritize the clinical validation of antioxidant therapy to complement the existing evidence base.

While a systematic framework for antioxidant therapy in vitiligo is currently lacking, available evidence supports its substantial therapeutic potential especially combined with phototherapies (**Table 1**). Oral vitamin E may represent a valuable adjuvant therapy of NB-UVB, preventing lipid peroxidation in the cellular membrane of melanocytes and increasing the repigmentation (74). Oral *Polypodium leucotomos extract* combined with NB-UVB improved repigmentation in a randomized, double-blind, placebo-controlled study, particularly at head and neck area (75). Oral α-lipoic acid, combined betamethasone injection, and NB-UVB therapy was safe and effective, and could promote the initial response of repigmentation (76). The combination of the antioxidant *Phyllanthus emblica*, vitamin E, and carotenoids with topical therapy and/or phototherapy has been shown to promote more significant repigmentation in the head, neck, and trunk of patients with vitiligo, while reducing both inflammation and the progression of skin lesions (77). A network meta-analysis in 2025, including 27 randomized controlled trials (RCTs) with a total of 2417 skin lesions, showed that for achieving ≥50% repigmentation, the surface under the cumulative ranking curve (SUCRA) score of phototherapy combined with antioxidants was as high as 87.7, which was much higher than that of phototherapy combined with glucocorticoids (69.6) and phototherapy combined with calcineurin inhibitors (52.5), while the score of phototherapy alone was only 3.1 (78). When achieving repigmentation of ≥75%, phototherapy combined with antioxidants also ranked first with 89.0 SUCRA score. An oral antioxidant pool containing α-lipoic acid, vitamins C and E and polyunsaturated fatty acids, given before and during NB-UVB treatment, significantly improved repigmentation and reduced markers of oxidative stress compared with NB-UVB alone (79). An RCT involving 50 patients demonstrated that oral gliadin-protected SOD combined with NB-UVB therapy resulted in greater improvement in Vitiligo Extent Score (VES) compared to placebo combined with NB-UVB (80). These data indicate that combined antioxidant therapy has great application prospects in vitiligo. Combined antioxidant therapy can have a synergistic effect with other treatment methods. The mechanism is related to that ultraviolet therapy for vitiligo has a double-edged sword effect, and ultraviolet radiation (UVR) induces the production of ROS, the accumulation of ROS can trigger oxidative stress, leading to lipid peroxidation, protein modification and DNA damage (81). This evidence highlights the heterogeneity in antioxidant type, dose and duration across included studies, and remains limited by small sample sizes, inconsistent outcome measures and short follow-up. Importantly, most studies enrolled unselected patients with stable or progressive non-segmental vitiligo rather than refractory and recurrent vitiligo.

**Table 1:** Key clinical trials and meta-analyses of antioxidant combination therapies in vitiligo.

Study and reference	Design and size	Intervention	Main result	Limitation
Elgoweini 2009 (74)	Randomized study; n = 24, 20 completed	Vitamin E + NB-UVB vs NB-UVB	Preventing lipid peroxidation of melanocytes and increasing the effectiveness of NB-UVB	The number of patients is small
Middelkamp-Hup 2007 (75)	Randomized double-blind placebo-controlled study; n = 50, 49 completed	*Polypodium leucotomos* capsules + NB-UVB vs placebo + NB-UVB	Increasing repigmentation in the head and neck area	Anatomic site specific
Li 2016 (76)	Randomized study; n = 50; non-segmental and progressive vitiligo	α-lipoic acid vs placebo, both received betamethasone injection + NB-UVB	Accelerating the initial response of repigmentation	No durable incremental benefit
Colucci 2015 (77)	Comparative controlled study; n = 130	*Phyllanthus emblica* + vitamin E + carotenoids treated with a comparable topical therapy and/or phototherapy	Increasing the effectiveness of other vitiligo treatments	Nonrandomized treatment background
Dell’Anna 2007 (79)	Double-blind placebo-controlled trial; n = 35, 28 completed	α-lipoic acid + vitamins C and E + polyunsaturated fatty acids vs placebo, with 2 months before and for 6 months during the NB-UVB treatment	Improving the clinical effectiveness of NB-UVB and reducing vitiligo-associated oxidative stress	Most of the lesions over the hands and feet showed mild to no response
Fontas 2021 (80)	Prospective randomized placebo-controlled study (Clinicaltrial.gov registration: NCT03941808); n = 50; 92% of patients failed to respond to at least one previous line of treatment, and most patients had stable disease	Gliadin-protected SOD + NB-UVB vs placebo + NB-UVB	Useful add-on to phototherapy in the treatment of vitiligo patients	Lack of measuring SOD levels at baseline and at 24 weeks
Luo 2025 (78)	Network meta-analysis; 27 randomized controlled trials of combined phototherapy with topical therapy in vitiligo	Topical therapies combined with phototherapy included corticosteroids, calcineurin inhibitors, vitamin D analogs, antioxidants, or fractional CO_2_ laser	For achieving ≥50% repigmentation, the top three combination therapies included phototherapy combined with antioxidants (SUCRA 87.7); For ≥75% repigmentation, the leading therapies included phototherapy combined with antioxidants (SUCRA 89.0)	Available data were insufficient; heterogeneity could not be entirely eliminated

Refractory or recurrent vitiligo patients were not specifically selected. No study supported that antioxidant therapy can treat vitiligo independently.

NB-UVB, narrowband ultraviolet B; SOD, superoxide dismutase; SUCRA, surface under the cumulative ranking curve.

Collectively, available evidence supports four potential roles for antioxidant therapy within combination therapeutic approaches including limitation of oxidative injury, reduction of DAMPs release and innate immune activation, improvement of the combination therapies efficacy, and decrease of oxidative triggers during maintenance after repigmentation. Further basic research and clinical trials are still required to provide evidence for antioxidant therapy in refractory and recurrent vitiligo.

## 5 A proposed clinical-mechanistic stratification framework

Refractory vitiligo is not the official classification of vitiligo in the international consensus, which is more often used as a clinical description (24). Recurrent vitiligo is defined as loss of pigmentation in previously repigmented lesions whether repigmentation occurred spontaneously or following treatment, and three months is the appropriate time frame for defining recurrence (82). Since poor treatment responses are often related to heterogeneous biological mechanisms, it is necessary to combine mechanism-based patient stratification with traditional clinical assessments. Clinical clustering and personalized medicine studies have demonstrated significant differences among patients with non-segmental vitiligo (NSV) in terms of disease activity, anatomical distribution, severity, prognosis, and treatment requirements (69, 83). Recent reviews on refractory vitiligo have further highlighted the persistent immune destruction, insufficient melanocyte regeneration, and chronic oxidative stress (24, 84). Based on these findings, we propose a clinical-mechanistic framework for generating research hypotheses, which includes four potential treatment-resistant phenotypes and one recurrent phenotype. These phenotypes are not mutually exclusive, and a single patient may present multiple phenotypes simultaneously, and the dominant underlying mechanisms may also vary depending on the disease course and treatment stage (**Table 2**).

**Table 2:** Proposed clinical-mechanistic phenotypes of refractory and recurrent vitiligo.

Phenotype	Clinical features	Dominant proposed mechanism	Candidate biomarkers	Current treatment	Potential role of antioxidants	References
Active-progression refractory	Continued and rapid progression or enlargement of lesions despite treatment; confetti-like lesions, trichrome, poorly defined borders, Koebner	Chronic oxidative stress, immune activation and amplification	TAC, MDA, 8-OHdG, CXCL9/10 (exploratory only)	Anti-inflammatory treatment, phototherapy	Potential adjunct (unvalidated)	(7, 10, 43, 82, 85)
IFN-amplification refractory	Persistent immune amplification	IFN-γ-JAK-STAT-CXCL9/10-CXCR3 pathway	CXCL9/10, IFN-γ signature (exploratory only)	Immune-targeted treatment, JAKi, phototherapy	Potential adjunct (unvalidated)	(56–61, 71, 86–90)
Site-specific refractory	Stable acral, joint, mucosal lesions; limited follicular or epidermal melanocyte reserves	Anatomical resistance and limited melanocyte reserve	Melanocyte or follicular reserve measures	Surgical or cellular transplantation	Low priority and no direct evidence	(24–28, 91)
Phototherapy-unresponsive refractory	Non-responding vitiligo lesions to phototherapy	Heterogeneous: melanocyte reserve, cellular senescence, chronic oxidative injury	Melanocyte reserve measures, cellular senescence markers, immune and redox markers (research only)	Consider combination or surgery for stable lesions (unvalidated)	Investigational adjunct	(75, 79, 80, 84, 92–99)
Recurrent vitiligo	Loss of pigmentation in previously repigmented lesions whether repigmentation occurred spontaneously or with treatment (international consensus)	IL-15/CD122-TRM-mediated local immune persistence	CD69/CD103, CD49a, CD122, IL-15, CXCL9/10 (tissue/exploratory/research only)	Topical tacrolimus	Exploratory adjunct; no evidence demonstrates that antioxidants eliminate TRM cells or prevent recurrence	(31–33, 35, 72, 73, 82, 100)

Phenotypes are overlapping and hypothesis-generating. Existing clustering and personalized medicine studies support clinically meaningful heterogeneity in vitiligo but do not directly validate the five proposed phenotypes.

TAC, total antioxidant capacity; MDA, malondialdehyde; 8-OHdG, 8-hydroxy-2-deoxyguanosine; CXCL, C-X-C motif ligand; IFN, interferon; JAK, Janus kinase; STAT, signal transducer and activator of transcription; CXCR, C-X-C motif chemokine receptor; JAKi, JAK inhibitor; IL, interleukin; TRM, tissue-resident memory T cells.

Active-progression refractory phenotype (phenotype 1) is characterized by continued and rapid progression or sudden enlargement of lesions despite adequate treatment. Itches, confetti-like depigmentation, trichrome vitiligo or poorly defined borders, and presence of Koebner phenomenon are associated with disease progression (82, 85). Experimental evidence indicates that oxidative stress-induced release of DAMPs, can promote DCs maturation and activation, chemokine production and CD8^+^ T cell recruitment (7, 10, 43). This phenotype is therefore hypothesized to involve persistent stress induced innate immune activation and early amplification. Antioxidant therapy may be biologically possible as an adjunct in active-progression refractory phenotype characterized by chronic oxidative stress and innate immune activation.

IFN-amplification refractory phenotype (phenotype 2) is characterized by persistent immune amplification through the IFN-γ-JAK-STAT-CXCL9/10-CXCR3 pathway. In the murine vitiligo model and clinical studies, IFN-γ signatures, increased CXCL9/10 and CXCR3 expression, together with disease reversal following disruption of this pathway in experimental models, provide strong mechanistic support for this circuit (86–90). Clinical evidence from JAKi studies further supports the therapeutic relevance of this pathway in NSV (56–61, 71). Elevated IFN-γ, CXCL9/10 may be a novel biomarker in this phenotype activity and guiding the treatment, although validated clinical assays and diagnostic thresholds are not currently available. In this phenotype, antioxidant therapy may reduce oxidative burden, but direct control of IFN immune signaling remains the principal therapeutic objective.

Site-specific refractory phenotype (phenotype 3) is characterized by clinically stable but poorly repigmentation lesions in which limited follicular or epidermal melanocyte reserves represent a major therapeutic constraint (24–28). Acral vitiligo and lesions over joints, mucosal sites are usually difficult to treat, supporting the importance of anatomical site and limited follicular melanocyte reserve (24, 91). Surgical or cellular transplantation may therefore be considered in appropriately selected stable lesions that remain unresponsive to adequate treatment (24). In site-specific refractory lesions, the role of antioxidant therapy is uncertain and probably low priority.

Phototherapy-unresponsive refractory vitiligo (phenotype 4) is an exploratory phenotype characterized by non-responding vitiligo lesions to phototherapy (84, 92, 93). In these lesions, the number of proliferative and differentiated melanocytes is low, and exhibit increased number of senescent keratinocytes and of cytotoxic CD8^+^ T cells. Its mechanisms are likely heterogeneous and may include cellular senescence related to ROS, low functional melanocyte reserve, inadequate melanocyte activation, altered melanogenic and cell-survival signaling, and excessive and chronic oxidative injury (84, 92, 94–96). Antioxidant treatment remains an investigational adjunct because clinical trials combining antioxidant therapy with phototherapy have produced inconsistent results, indicating that oxidative stress cannot yet be regarded as an established cause of phototherapy resistance (75, 79, 80, 97–99). In phototherapy-unresponsive lesions, antioxidant treatment remains investigational because oxidative stress has not been established as the dominant cause of phototherapy resistance.

Recurrent vitiligo (phenotype 5) should be distinguished from refractory vitiligo. According to the international consensus, recurrent vitiligo is defined as loss of pigmentation in previously repigmented lesions whether repigmentation occurred spontaneously or with treatment (82). Melanocyte-specific CD8^+^ TRM cells exist in both the lesions and the nonlesional skin of patients with vitiligo, and the long-term survival and functional maintenance of TRM cells in the skin depend on the IL-15 signaling pathway (31, 33, 35). IL-15/CD122 signaling provides a biological mechanism for recurrence, although the strongest evidence for disease reversal following IL-15/CD122 blockade currently derives from experimental results (31). In maintenance studies, twice-weekly topical tacrolimus may reduce renewed depigmentation, while continued ruxolitinib improves the probability of maintaining facial repigmentation in previous responders (32, 72, 73, 100). No clinical evidence demonstrates that antioxidants eliminate TRM cells or prevent recurrence, and antioxidant treatment should therefore remain an exploratory adjunct.

This framework is intended as a hypothesis-generating model rather than a validated classification system. Existing clustering and personalized medicine studies support clinically meaningful heterogeneity in vitiligo but do not directly validate the five proposed phenotypes (20, 22, 69, 82–84). For clinical and research application, the proposed framework may be approached sequentially based on current clinical assessment principles (20, 21, 24, 69, 83). Clinical features of vitiligo should first be characterized by disease activity, treatment exposure, adherence, anatomical site, and previous treatment response, and previous repigmentation followed by renewed depigmentation (20, 21, 24, 82). Molecular biomarkers may subsequently be incorporated as exploratory markers rather than diagnostic criteria. For hypothesis-driven experiments, persistent oxidative stress, DAMP release, IFN-γ, and CXCL9/10 could be used to induce active-progression and IFN-amplification refractory vitiligo model. Treatment withdrawal during a repigmentation phase or combing it with IL-15 signaling may induce recurrent vitiligo model. However, these models are currently based on the proposed clinical-mechanistic stratification framework, and have not yet been fully implemented in practice, requiring further validation. Importantly, this approach links hypothesis-generating phenotypes to biological mechanisms without implying that current mouse models fully reproduce refractory or recurrent human disease.

Collectively, future clinical trials and observational studies investigating refractory or recurrent vitiligo should report disease subtype, disease activity, site-specific Vitiligo Area Scoring Index (VASI) measurement, treatment history and duration, and previous repigmentation for recurrence. If possible, potential oxidative stress markers, IFN-related chemokines and cytokines, and tissue-memory signatures may also be assessed as exploratory biomarkers (42, 88–90, 101). It is necessary to conduct prospective biomarker-based stratification studies, providing a basis for guiding precision therapies and combination treatment strategies for refractory and recurrent vitiligo.

## 6 Challenges and prospects

Oxidative stress also contributes to the pathogenesis of several inflammatory skin disorders, including allergic contact dermatitis, varicose ulcers, and skin carcinomas (102). However, this shared mechanism has not made antioxidant therapy a standard of care for these skin disorders. Their established management remains disease-specific, including appropriate topical anti-inflammatory therapy for contact dermatitis (103), compression therapy and appropriate wound care for varicose ulcers (104), and UV-risk prevention for skin carcinomas (105). Experience from other skin disorders supports the biological relevance of redox dysregulation but also indicates the limitations of directly translating mechanistic oxidative stress observations into clinical antioxidant treatment. The same caution should be applied to vitiligo that redox abnormalities are biologically relevant but are not sufficiently specific to justify antioxidant treatment without standard therapy.

The lack of specific and unified clinical standards for antioxidant therapy makes it hard to administer medication. Although an increasing number of studies support the value of antioxidant therapy in vitiligo, none of the consensuses have provided standardized protocols in terms of the specific types, dosages, treatment courses, administration routes and applicable patient groups of antioxidants. At present, the clinical positioning of antioxidant therapy is still adjunctive rather than an independent core treatment approach. A large-scale prospective questionnaire study from China shows that 90.7% support the theory of oxidative stress-induced vitiligo, yet only 11.8% of the patients frequently treat vitiligo with antioxidants (106). The data show that the uncertainty of the efficacy of antioxidants (84.2%) is the main reason, followed by controversial evidence of the efficacy of antioxidants in dermatology textbooks or literature (74.1%) and insufficient knowledge of antioxidants (72.7%) (106). The guiding value of the existing evidence remains limited, especially as large-scale clinical data are needed to verify the efficacy of antioxidants and incorporate them into official guidelines. This highlights the limited guiding value of existing evidence, underscoring the need for large-scale clinical data to confirm the efficacy of antioxidants, and for consensus-based standards of antioxidant therapy that can be incorporated into clinical guidelines.

It is necessary to clarify the patients suitable for combined antioxidant therapy and the corresponding antioxidant therapy. Not all patients with vitiligo can benefit equally from antioxidant treatment. Existing evidence suggests that the predictors associated with higher antioxidant efficacy included advanced or rapid progression, moderate or moderate-to-severe vitiligo, age, segmental vitiligo (106). In addition, molecular-level biomarkers are also needed to achieve precise screening. Candidate markers could include Nrf2 gene polymorphism and the expression levels of its downstream target genes, baseline serum antioxidant enzyme activity, systemic oxidative damage indicators such as 8-OHdG, and immune biomarkers including CXCL9, CXCL10, IFN-γ and IL-15-related signatures. By integrating these markers, it is possible to pre-determine whether a patient is oxidative stress-sensitive, and based on this, different types of antioxidants can be selected. In the future, a clinical predictive scoring system should be established to guide individualized medication decisions.

The evaluation system for the efficacy of antioxidant therapy is not yet well-established in refractory and recurrent vitiligo. Most current studies take the short-term repigmentation rate (such as ≥50% repigmentation) as the primary endpoint, ignoring indicators that better reflect the long-term value of antioxidant treatment, such as the duration of repigmentation maintenance, annual recurrence rate, and dynamic changes in oxidative stress markers. These evaluation indicators cannot reveal whether antioxidant therapy truly improves the oxidative stress state. More crucially, the correlation between the changes of these markers and the repigmentation has not been systematically verified.

Targeted delivery of antioxidants and research directions of new antioxidants should be considered. Traditional antioxidant treatments are confronted with the limitations of insufficient targeting and a single mechanism. Li et al. introduced a novel therapeutic agent called polydopamine (PDA)-JAKi, which is capable of both reducing oxidative stress and inhibiting T cell activation (107). This serves as a good paradigm for the development of novel antioxidants. In terms of new antioxidants, the research direction is shifted from traditional free radical scavengers to Nrf2 activators and ferroptosis inhibitors. In other respects, polyphenolic compounds have demonstrated therapeutic potential through multiple mechanisms such as antioxidant activity, immune regulation, and melanocytogenesis promotion. Future research can focus on optimizing the nanoparticle-based formulations, mitochondria-targeted antioxidants, and drug delivery system to enhance the application of antioxidants in the treatment of vitiligo (108, 109).

Several novel therapeutic approaches for vitiligo are associated with oxidative stress. In melanocytes, the mammalian target of rapamycin (mTOR) signaling pathway is crucial for preventing oxidative stress (110). The mTOR inhibitor rapamycin can significantly enhance melanogenesis and may be associated with oxidative stress and metabolic dysfunction in melanocytes of vitiligo patients. A phase 2 clinical trial is evaluating daily topical rapamycin for the treatment of vitiligo (NCT05342519, https://www.clinicaltrials.gov/; registered April 22, 2022). In vitiligo, oxidative stress impairs the enzymes responsible for prostaglandin E2 (PGE2) synthesis (84). Both PGF2α and PGE2 intradermal injections can be considered the effective treatments for stable vitiligo, with no reported adverse effects and high patient satisfaction (111). The clinical trial NCT07016113 (https://www.clinicaltrials.gov/; registered June 11, 2025) aims to evaluate the efficacy of 0.005% latanoprost gel (prostaglandin F2α analog) for non-segmental vitiligo with 308 nm excimer phototherapy. The therapeutic potential of phosphodiesterase-4 (PDE-4) inhibitors in refractory vitiligo lies in their ability to simultaneously stimulate melanin production while reducing inflammation and oxidative stress (84). A phase 2 clinical trial evaluating PF-07038124 (a PDE-4 inhibitor) has been completed (NCT05298033, https://www.clinicaltrials.gov/; registered March 28, 2022). Melanocytes under oxidative stress aberrantly produce HSP70i, which stimulates plasmacytoid dendritic cells to produce IFN-α, thereby inducing the expression of the chemokines CXCL9 and CXCL10 in keratinocytes in vitiligo (7, 112). For this pathway, DNA-based therapeutic approaches encoding HSP70iQ435A promoted repigmentation and a monoclonal antibody targeting IFN-α receptor 1 anifrolumab (NCT05917561, https://www.clinicaltrials.gov/; registered June 26, 2023) is currently under development for clinical testing in vitiligo patients (113, 114). Nociceptor neuron-derived calcitonin gene-related peptide (CGRP) strengthens cDC1-CD8^+^ T cell interactions to promote vitiligo (115). Under oxidative stress conditions, the CGRP signaling pathway impairs the expansion of regulatory T cells (Tregs), thereby further exacerbating immune imbalance (116). Combination of mouse model experiments and a pilot study suggest that CGRP receptor antagonist rimegepant represents a safe and targeted drug candidate for vitiligo treatment (115). Platelet-rich plasma (PRP) modulates cytokine expression, enhances tissue repair, and regulates oxidative stress, demonstrating anti-inflammatory and immunomodulatory effects in various inflammatory diseases (117). Recent evidence suggests that PRP may represent a promising therapeutic strategy for refractory vitiligo by promoting the differentiation of dermal melanocyte progenitors into functional melanocytes through the IGFBP-2/RhoA/YAP axis (118). An increasing number of therapies are associated with oxidative stress in vitiligo, highlighting its significance and value in the treatment.

Prospective exploration of the combination of antioxidant therapy with JAKi and IL-15-targeted therapy demonstrates broad promise. Refractory and recurrent vitiligo are not caused by excessive ROS or abnormal immune pathways alone, but rather by the combined effect of oxidative damage, immune inflammation, insufficient melanocyte reserves and TRM immune memory. JAKi block IFN-γ-driven JAK-STAT signaling and reduce CD8^+^ T cell infiltration, achieving clinically meaningful repigmentation (71). However, they do not address the oxidative injury that continuously fuels antigen release and immune activation. IL-15 antibody therapy also faces the same problem. Therefore, a single treatment often fails to simultaneously address the four issues of promoting repigmentation, anti-inflammation, preventing recurrence, and stabilizing the microenvironment. Future studies are warranted to investigate the combination of antioxidants with diverse treatment strategies.

## 7 Conclusion

Vitiligo is an autoimmune pigmentary disorder, and oxidative stress serves as a critical factor in its pathogenesis, linking melanocyte dysfunction with innate and adaptive immune activation, thereby contributing to the disease progression. The role of oxidative stress may lie in affecting melanocyte activity, promoting the release of immunogenic signals, and enhancing the inflammatory response that sustains an immune response against melanocytes, and cannot fully explain the mechanisms underlying vitiligo resistance and recurrence. Refractory and recurrent vitiligo represent heterogeneous and partially overlapping clinical issues that require different therapeutic approaches rather than a uniform management strategy.

IFN-γ-JAK-STAT-CXCL9/10-CXCR3 signaling is closely associated with the active immune amplification process, whereas IL-15-dependent TRM cells provide a biological explanation for the rapid reactivation of the local immune response following resolution of inflammation, highlighting maintenance therapy as a mechanistically distinct therapeutic challenge. More and more novel investigational therapies are associated with oxidative stress in vitiligo, highlighting its significance and value in the treatment. However, treatment efficacy is also significantly affected by factors such as the anatomical sites, white hair, melanocyte reserve. Current evidence does not support antioxidant therapy as a single treatment, or the therapeutic approach eliminates TRM cells or prevents disease recurrence. Existing anti-inflammatory, repigmentation, and maintenance treatments should continue to serve as the foundation for disease management, and antioxidant therapy may serve as an adjunctive treatment in vitiligo.

Future research should clearly develop the clinical definitions for refractory vitiligo and recurrent vitiligo, and implement patient stratification based on disease activity levels, biomarkers, and other factors. Integration of oxidative stress markers with IFN-related chemokines and immune-memory signatures may help identify the patient stratification, disease course, and treatment stage. This approach, combined with patient stratification, provides the potential to transform antioxidant therapy from an adjunctive treatment into a precision supportive therapy targeting specific phenotypes in vitiligo. However, before applied to support precision antioxidant combination therapy, they still require validation through prospective studies including basic research and clinical trials.
